# Editorial: New applications of biological and computational neural networks in brain imaging

**DOI:** 10.3389/fnins.2025.1590069

**Published:** 2025-04-11

**Authors:** Prasanna Parvathaneni

**Affiliations:** Independent Researcher, Bethesda, MD, United States

**Keywords:** computational methods, neuroscience, brain imaging, neurological disorders, functional connectivity

In the rapidly evolving field of neuroscience, the fusion of biological insights with computational neural networks has opened new frontiers in brain imaging. This integration is not merely enhancing the precision of diagnostics and expanding therapeutic strategies; it is fundamentally transforming our approach to understanding and treating neurological disorders. As we stand on the verge of these technological advancements, it is imperative to consider their broader implications not only for individual patient care but also for societal norms, ethical standards, and healthcare economics. This editorial examines transformative technologies in computational neuroscience, emphasizing their pivotal role in revolutionizing the treatment of neurological disorders, with profound implications for both personalized care and widespread clinical application.

One of the most promising applications of computational brain imaging lies in neuromodulation therapies, particularly in deep brain stimulation (DBS) for Parkinson's disease (PD). Li Z. et al. introduce delta functional controllability as a novel biomarker to predict motor outcomes of DBS in PD. Using support vector regression, the study demonstrates that preoperative functional controllability can serve as an indicator of DBS response, enhancing personalized treatment decisions. The findings reveal that distinct focal regions within the cortico-striato-thalamo-cortical motor loops are involved in STN-DBS and GPi-DBS, supporting the feasibility of brain-controllability-based biomarkers for mechanistically understanding and predicting DBS effects for clinical decision-making. This study advances our knowledge of Parkinson's disease-related brain network dysfunction and proposes broader implications for neurological disorders. By integrating network control theory with functional magnetic resonance imaging (fMRI), it contributes to predictive modeling and neuromodulation techniques. This approach mitigates surgical risks, financial burdens, and enhances precision in the selection of DBS candidates and the optimization of therapeutic outcomes.

Beyond movement disorders like PD, network analysis also provides significant insights into epilepsy, another neurological condition characterized by abnormal connectivity. Li Y. et al. investigates generalized tonic-clonic seizures (GTCS) in children, examining how these seizures affect static (sFC) and dynamic functional connectivity (dFC) using resting-state fMRI. Unlike prior studies focused on adults, this research uniquely explores pediatric network disruptions and identifies two distinct brain states: State 1 which exhibits strong connectivity but is reduced in GTCS, State 2 is characterized by weaker connectivity but increased in GTCS. These findings suggest that the duration of epilepsy correlates with changes in dFC, indicating progressive network reorganization over time. These insights enhance our understanding of how GTCS disrupts neural communication, particularly within visual, motor, attention, and default mode networks. The study not only highlights potential biomarkers for disease progression and personalized treatment but also underscores the value of leveraging computational techniques like independent component analysis (ICA) and k-means clustering to assess static and dynamic functional network connectivity in children with GTCS using resting-state fMRI. The research contributes significantly to computational neuroscience by providing deeper insights into the biological network dysfunctions associated with epilepsy. These findings help to establish a foundation for future applications that may incorporate neural network models to better understand, diagnose, and treat neurological conditions based on complex brain imaging data. Such innovations are pivotal for early diagnosis, treatment response prediction, and devising neuromodulation strategies, bridging computational neuroscience with real-world clinical applications.

Another significant breakthrough from Luo et al. utilizes network analysis to explore the differences in the distribution of triggers among resting-state networks in patients with Juvenile Myoclonic Epilepsy (JME). By employing a small-world network model, the research systematically maps and visualizes resting-state connectivity and analyzes white matter tract restrictions. This approach identifies that the thalamus, cerebellum, basal ganglia, supplementary motor area, visual cortex, and prefrontal cortex as core brain regions implicated in the pathophysiology of JME. The findings underscore reduced centrality in the prefrontal and visual cortices, potentially explaining why only some patients develop seizures in response to visual stimuli or cognitive loads. By systematically mapping and reducing nodes in these network analyses, the study highlights how changes in functional connectivity correlate with epilepsy symptoms, providing a deeper understanding of the biological network dysfunctions associated with JME. The findings suggest potential targets for neuromodulation therapies and personalized interventions. Insights into white matter fiber tract connectivity highlight crucial structural-functional relationships for advancing epilepsy treatment. This research exemplifies how computational techniques in brain imaging, particularly systematic network analysis, can offer significant insights into the complex dynamics of neurological disorders like epilepsy. The methodologies applied here could serve as a model for future studies aiming to leverage computational neural networks for diagnostic and therapeutic purposes in various neurological conditions.

Accurate EEG signal extraction is crucial to computational brain imaging, particularly in addressing challenges related to noise reduction and artifact management. Xiong et al. introduce an efficient solution with development of a Dual-Pathway Autoencoder (DPAE), which significantly enhances EEG signal clarity, essential for neurological diagnosis and treatment. This innovative model integrates a dual-pathway structure with blind source separation techniques, enabling effective artifact removal without requiring prior knowledge of noise distribution. The DPAE model, validated on benchmark datasets, outperforms existing deep learning denoising methods, particularly in reducing eye (EOG) and muscle (EMG) artifacts. Its lightweight design makes it ideal for real-time applications in portable brain-computer interfaces, expanding the potential for at-home neurological monitoring and telemedicine applications.

Collectively, these studies push the boundaries of computational and biological methods in brain imaging. By enhancing diagnostic accuracy and paving the way for personalized medicine, they enable interventions tailored to individual brain networks. As computational models grow more sophisticated, their integration into clinical workflows will transform neurological care, offering unprecedented precision in diagnostics, therapeutic planning, and neuromodulation strategies. The synergy between computational models and clinical applications signifies a paradigm shift, transforming our approach to neurological healthcare. Moving forward, it is essential for researchers, clinicians, and policymakers to collaborate closely to develop and implement these technologies responsibly, ensuring innovation is balanced with accessibility, ethical integrity, and patient-centered care. Together, we can leverage these advancements to treat neurological disorders and shape a future where technology and human health progress together, delivering care as precise and nuanced as the neural networks we study.

